# Is serum HMGB1 a biomarker in ANCA-associated vasculitis?

**DOI:** 10.1186/ar4284

**Published:** 2013-09-04

**Authors:** Alexandre Wagner Silva de Souza, Johanna Westra, Johan Bijzet, Pieter C Limburg, Coen A Stegeman, Marc Bijl, Cees GM Kallenberg

**Affiliations:** 1 Department of Rheumatology and Clinical Immunology, University Medical Center Groningen, University of Groningen, Hanzeplein 1, 9713 GZ Groningen, the Netherlands; 2 Rheumatology Division, Universidade Federal de São Paulo, Escola Paulista de Medicina (Unifesp/EPM), Rua Botucatu 740, 04023-900 São Paulo, Brazil; 3 Department of Laboratory Medicine, University Medical Center Groningen, University of Groningen, Hanzeplein 1, 9713 GZ Groningen, the Netherlands; 4 Department of Internal Medicine, Division of Nephrology, University Medical Center Groningen, University of Groningen, Hanzeplein 1, 9713 GZ Groningen, the Netherlands; 5 Department of Internal Medicine and Rheumatology, Martini Hospital, Van Swietenlaan 1, 9728 NT Groningen, the Netherlands

## Abstract

**Background:**

Antineutrophil cytoplasmic antibody (ANCA)-associated vasculitides (AAV) are systemic inflammatory disorders that include granulomatosis with polyangiitis (GPA), microscopic polyangiitis (MPA), Churg-Strauss syndrome and renal limited vasculitis (RLV). Extracellular high-mobility group box 1 (HMGB1) acts as an alarmin and has been shown to be a biomarker of disease activity as well as an autoantigen in systemic lupus erythematosus (SLE) and, possibly, in AAV. This study aims to assess antibodies against HMGB1 and HMGB1 levels as biomarkers for AAV disease activity and predictors of relapsing disease.

**Methods:**

AAV patients with active disease and healthy controls (HC) were evaluated for anti-HMGB1 antibodies while serum HMGB1 levels were measured longitudinally in AAV patients at presentation, during remission, prior to and at relapses.

**Results:**

HMGB1 levels were similar between AAV patients at presentation (*n *= 52) and HC (*n *= 35) (2.64 ± 1.80 ng/ml vs. 2.39 ± 1.09 ng/ml; *P *= 0.422) and no difference regarding HMGB1 levels could be found among AAV disease subsets (GPA: 2.66 ± 1.83 ng/ml vs. MPA: 3.11 ± 1.91 ng/ml vs. RLV: 1.92 ± 1.48 ng/ml; *P *= 0.369). AAV patients with renal involvement had lower HMGB1 levels than patients without renal involvement at presentation (2.35 ± 1.48 ng/ml vs. 3.52 ± 2.41 ng/ml; *P *= 0.042). A negative correlation was observed between HMGB1 levels and 24-hour proteinuria (ρ = -0.361, *P *= 0.028). Forty-nine AAV patients were evaluated for HMGB1 levels during follow-up and no differences were observed between relapsing and nonrelapsing patients (*P *= 0.350). No significant increase in HMGB1 levels was observed prior to a relapse compared with the remission period and changes in HMGB1 levels were not associated with an increased risk for relapse in AAV. Positivity for anti-HMGB1 antibodies was low in patients with active AAV (three out of 24 patients).

**Conclusions:**

Serum HMGB1 levels at presentation are not increased and are lower in patients with renal involvement. Relapses are not preceded or accompanied by significant rises in HMGB1 levels and changes in HMGB1 levels are not related to ensuing relapses. Anti-HMGB1 antibodies are present in only a few patients in AAV. In contrast to SLE, HMGB1 is not a useful biomarker in AAV.

## Introduction

Antineutrophil cytoplasmic antibody (ANCA)-associated vasculitides (AAV) are primary systemic vasculitides affecting small and medium-sized vessels, and are associated with ANCA against proteinase 3 (PR3) and myeloperoxidase. AAV include granulomatosis with polyangiitis (GPA), microscopic polyangiitis (MPA), Churg-Strauss syndrome, and isolated pauci-immune necrotizing crescentic glomerulonephritis also designated as renal limited vasculitis (RLV) [1,2]. Disease relapses are common in AAV and occur in up to 60% of patients, especially in patients with GPA and PR3 ANCA [3-7]. Risk factors for relapses in AAV include the persistence of PR3 ANCA after induction of remission, upper and lower airway involvement, cardiovascular involvement, and chronic nasal carriage of *Staphylococcus aureus*, particularly strains that express the toxic shock syndrome toxin-1 superantigen gene [3,5,6,8]. A recent meta-analysis showed that the rise in ANCA titers or their persistence during remission is only modestly associated with an increased risk of relapses in AAV patients [9]. There is thus an unmet need for biomarkers predicting which AAV patient is prone to relapse.

High-mobility group box-1 (HMGB1) is a nuclear protein that binds DNA and modulates chromosomal architecture. Once released into the extracellular space, after cell death or upon activation, HMGB1 acts as a danger-associated molecular pattern or as an alarmin and stimulates inflammatory and immunological activities that include cytokine production, chemotaxis, cell proliferation, angiogenesis and cell differentiation. HMGB1 has to bind to the receptor for advanced glycation end-products (RAGE) and toll-like receptor (TLR)-2, TLR-4 and TLR-9 in order to exert its actions [10,11].

In systemic lupus erythematosus (SLE), serum HMGB1 has been shown to be a biomarker of disease activity, especially in patients with lupus nephritis. Moreover, patients with active lupus nephritis present higher HMGB1 levels in urine compared with SLE patients without active nephritis and with controls [12-14]. Furthermore, levels of antibodies to HMGB1 are higher in patients with active SLE than in patients with quiescent disease and in controls [13]. In AAV, a cross-sectional study showed increased serum levels of HMGB1 in patients with active GPA [15]. In addition, one study found an association with granulomatous manifestations and another with biopsy-proven renal involvement [16,17].

Until now, HMGB1 levels have not been evaluated longitudinally as a biomarker of disease activity or as a predictor of ensuing relapses in patients with AAV. The aims of this study were to evaluate whether serial levels of HMGB1 reflect changes in disease activity and/or predict the occurrence of relapses, and to analyze whether AAV patients have antibodies to HMGB1.

## Materials and methods

### Patients

Patients on follow-up at the University Medical Center Groningen with a diagnosis of AAV, including GPA, MPA, and RLV, were eligible for the study. Patients had a clinical diagnosis of GPA or MPA according to the European Medicines Agency algorithm [18]. Patients with isolated renal involvement, ANCA positivity and biopsy-proven pauci immune necrotizing glomerulonephritis were classified as RLV. ANCA tests were performed in all patients by indirect immunofluorescence using ethanol-fixed neutrophils, while ANCA specificity for PR3 or myeloperoxidase was assessed by enzyme-linked immunosorbent assay (ELISA). To assess whether HMGB1 levels are increased in active disease, 52 AAV patients were included at presentation; characteristics are presented in Table 1. Additionally, 49 out of 52 AAV patients with sufficient follow-up data were included in a longitudinal analysis and were evaluated during a mean period of 54.4 ± 17.6 months. Thirty-five age-matched and sex-matched healthy controls (HC) were evaluated for HMGB1 levels in the study as well. All patients and HC gave informed consent. The study was conducted according to the ethical guidelines of the University Medical Center Groningen, approved by the ethical committee of the University Medical Center Groningen, and in accord with the Declaration of Helsinki.

**Table 1:** Baseline features at presentation and therapy in 52 patients with antineutrophil cytoplasmic antibodies-associated vasculitis

Variable	Result
Diagnosis	
Granulomatosis with polyangiitis	33 (63.5)
Microscopic polyangiitis	11 (21.2)
Renal limited vasculitis	8 (15.4)
ANCA	
Proteinase 3 ANCA	30 (57.7)
Myeloperoxidase ANCA	22 (42.3)
Disease activity	
Median BVAS	15.0 (12.0 to 23.5)
Median C-reactive protein level (mg/l)	37.0 (11.5 to 81.5)
Disease manifestations	
Renal involvement	39 (75.0)
Systemic manifestations	32 (61.5)
Ear, nose and throat involvement	28 (53.8)
Pulmonary involvement	22 (42.3)
Arthritis/joint pain	18 (34.6)
Peripheral neuropathy	15 (28.8)
Eye involvement	13 (25.0)
Cutaneous vasculitis	12 (23.1)
Pulmonary involvement	
Pulmonary nodules and/or infiltrates	12 (23.0)
Alveolar hemorrhage	6 (11.5)
Pleural effusion	2 (3.8)
Endobronchial lesion	1 (1.9)
Renal-related variables	
Median 24-hour proteinuria (g)	0.90 (0.55 to 1.60)
Hematuria (>10 RBC/HPF)	39 (75.0)
Median creatinine (μmol/l)	137.0 (80.0 to 350.0)
Mean creatinine clearance (ml/minute/1.73 m^2^)	65.7 ± 41.7
Dialysis dependent	8 (15.4)
Actual therapy	
Patients without treatment	27 (51.9)
Prednisolone and cyclophosphamide	13 (25.0)
Prednisolone only	7 (13.5)
Plasmapheresis	6 (11.5)
Mean number of plasmapheresis sessions	9.33 ± 1.50
Methotrexate	1 (1.9)

Data presented as *n *(%), median (interquartile range) or mean ± standard deviation. ANCA, antineutrophil cytoplasmic antibodies; BVAS, Birmingham Vasculitis Activity Score; HPF, high-power field; RBC, red blood cells.

AAV patients and HC were matched for age (58.8 ± 14.0 vs. 55.7 ± 11.7 years; *P *= 0.277) and gender (44.2% vs. 51.4% females; *P *= 0.510). Throughout the study, AAV patients were evaluated for HMGB1 levels, disease activity using the third version of the Birmingham Vasculitis Activity Score (BVAS) [19], ANCA status, C-reactive protein (CRP) levels, and therapy. Complete remission was defined as a BVAS of 0 in combination with a normal serum CRP level (<10 mg/l). Relapse was defined as the need to restart or intensify immunosuppressive therapy due to biopsy-proven or clinically suspected vasculitic disease activity. Anti-HMGB1 antibodies were tested in a randomly selected sample of AAV patients with active disease and data were compared with those in HC. In the longitudinal analysis, AAV patients were evaluated for HMGB1 serum levels at presentation, and twice during the remission period, namely at 3 months (interquartile range 3 to 6) and 11 months (interquartile range 6 to 12) after presentation. For relapsing AAV patients, HMGB1 levels were measured 2 months (interquartile range 1 to 3) prior to each relapse and at the moment of relapse. Relapsing and nonrelapsing AAV patients were compared regarding HMGB1 levels at baseline and during the remission period.

### ELISA for serum HMGB1

HMGB1 levels were assessed in AAV patients and HC using a commercial ELISA kit according to the manufacturer's instructions (Shino Test; Sagamihara, Kanagawa, Japan). Results of serum HMGB1 levels are expressed in nanograms per milliliter.

### ELISA for anti-HMGB1 antibodies

Anti-HMGB1 antibodies were tested in 24 AAV patients with active disease and 18 HC using an in house-developed ELISA described previously [13]. Sera from two patients with active SLE and high titers of anti-HMGB1 antibodies were used as positive controls. Briefly, Maxisorp polystyrene 96-well plates were coated with 50 μl/well recombinant HMGB1 (R&D Systems, Abingdon, UK) at 1 μg/ml in phosphate-buffered saline and incubated overnight at 4°C. Plates were then blocked with 5% bovine serum albumin in phosphate-buffered saline for 2 hours. Serum samples, diluted 1:50 in incubation buffer, were added to the plate (100 μl/well) and incubated for 2 hours at room temperature. After five washes, 100 μl horseradish peroxidase-conjugated goat anti-human IgG (SouthernBiotech, Birmingham, AL, USA) diluted 1:3,000 was added to each well and incubated for 1 hour at room temperature. After washing, bound antibodies were detected using 3,3',5,5'-tetramethylbenzidine dihydrochloride. The reaction was stopped with 2 M sulfuric acid and the absorbance was measured at 450 nm using a microplate spectrophotometer (Vmax; Molecular Devices, Sunnyvale, CA, USA). Results are expressed as optical density (OD) and anti-HMGB1 antibodies were considered positive if OD values were above the cutoff level of 0.435 obtained from the mean plus twice the standard deviation in 18 HC.

### Statistical analysis

Statistical analysis was performed using SPSS software version 20.0 and graphs were built using Graph Pad Prism version 3.02. Categorical variables were presented as the total number and percentage whereas continuous data were presented as the mean ± standard deviation when variables were normally distributed or as the median and interquartile range in case of non-normal distribution. Comparison between groups was performed using the chi-square test or Fisher's exact test for categorical variables and Student's *t *test or the Mann-Whitney U test for continuous data. One-way analysis of variance test was used for comparisons between three or more groups for numerical variables, and *post-hoc *analysis was performed with Dunnet's or Tukey's tests. Correlation between numerical data was calculated using Spearman's or Pearson's correlation coefficient when appropriate. Fluctuations in HMGB1 levels during follow-up in AAV patients were evaluated by Friedman's test and in cases of significance the Wilcoxon rank-sum test was used. Comparison between relapsing and nonrelapsing AAV patients regarding HMGB1 levels during follow-up was performed by generalized estimating equations. Cox proportional hazard models were built to analyze whether changes in HMGB1 levels were related to time of first relapse in AAV patients. Results are expressed as the hazard ratio and 95% confidence interval. The significant level accepted was 5% (*P *< 0.05).

## Results

### Baseline features of AAV patients

Characteristics of the 52 patients included in the study are presented in Table 1. All AAV patients were positive for anti-PR3 ANCA (*n *= 30) or anti-myeloperoxidase ANCA (*n *= 22) at presentation. Eight GPA patients (15%) had localized disease restricted to the upper and/or lower respiratory tract, eyes and/or ears, whereas active renal involvement was the most frequent disease manifestation at baseline. Most AAV patients (52%) were not on treatment at baseline evaluation, while among treated patients the combination of prednisolone and oral cyclophosphamide was most commonly prescribed (Table 1). In the patients already on immunosuppressive therapy, the median duration prior to baseline evaluation was 3.0 weeks (interquartile range 1.0 to 4.0). In this subgroup the median daily prednisolone dose was 60 mg while the median daily dose of oral cyclophosphamide was 150 mg. Although those patients were already under therapy at baseline evaluation, no difference regarding median BVAS could be found between treated and untreated patients (14.0 (12.0 to 23.0) vs. 15.0 (12.0 to 26.0); *P *= 0.491).

### HMGB1 levels at baseline

**Figure 1:**
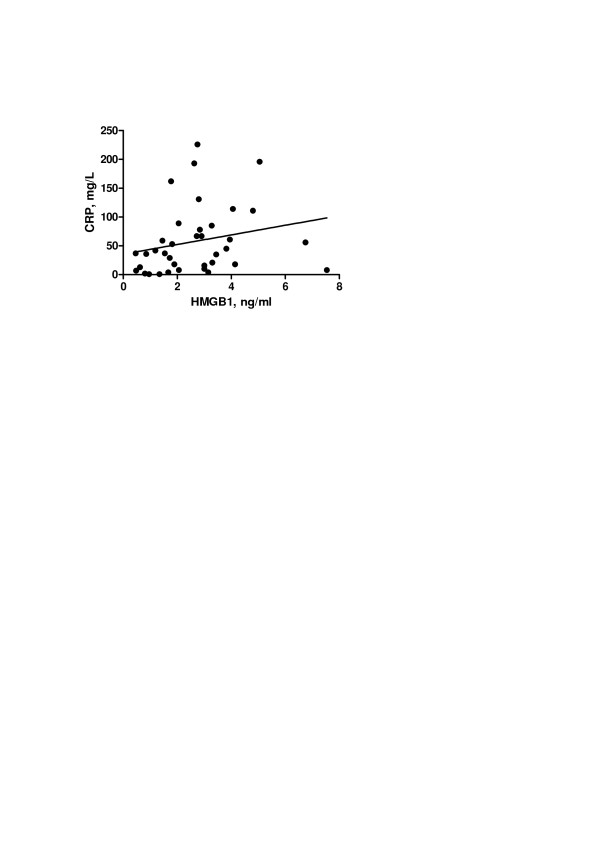
**Correlation between serum HMGB1 and C-reactive protein levels at presentation in AAV patients**. A significant positive correlation was found between serum high-mobility group box 1 (HMGB1) and C-reactive protein (CRP) levels in antineutrophil cytoplasmic antibodies-associated vasculitis (AAV) patients at presentation (ρ = 0.341; *P *= 0.039).

Mean HMGB1 levels were similar between AAV patients and HC (2.64 ± 1.80 ng/ml vs. 2.39 ± 1.09 ng/ml; *P *= 0.422) and no significant differences were found regarding mean HMGB1 levels among AAV disease subsets (GPA: 2.66 ± 1.83 ng/ml vs. MPA: 3.11 ± 1.91 ng/ml vs. RLV: 1.92 ± 1.48 ng/ml; *P *= 0.369). Although not significant, HMGB1 levels were higher in GPA patients with localized disease in comparison with those presenting generalized disease (3.14 (1.87 to 3.78) ng/ml vs. 1.84 (1.30 to 3.36) ng/ml; *P *= 0.240) as well as in AAV patients with pulmonary nodules and/or infiltrates compared with those presenting alveolar hemorrhage (3.60 ± 1.99 ng/ml vs. 2.09 ± 1.18 ng/ml; *P *= 0.107), but these differences did not reach statistical significance. Patients who were already under treatment for AAV at baseline had similar mean HMGB1 levels in comparison with those patients without immunosuppressive therapy (2.52 ± 1.58 ng/ml vs. 2.75 ± 2.01 ng/ml; *P *= 0.651) while lower HMGB1 levels were found in AAV patients who underwent sessions of plasmapheresis and/or dialysis prior to baseline evaluation of HMGB1 than in AAV patients without these therapies (1.84 ng/ml (1.55 to 2.79) vs. 2.63 ng/ml (1.22 to 3.88); *P *= 0.388), but this difference was not significant. A positive but weak correlation was found between serum HMGB1 and CRP levels (ρ = 0.341; *P *= 0.039) (Figure 1), while no correlation was found between serum HMGB1 levels and BVAS (ρ = -0.019; *P *= 0.896), cytoplasmic ANCA titers (ρ = -0.208; *P *= 0.271) or perinuclear ANCA titers (ρ = 0.054; *P *= 0.813) at presentation.

**Figure 2:**
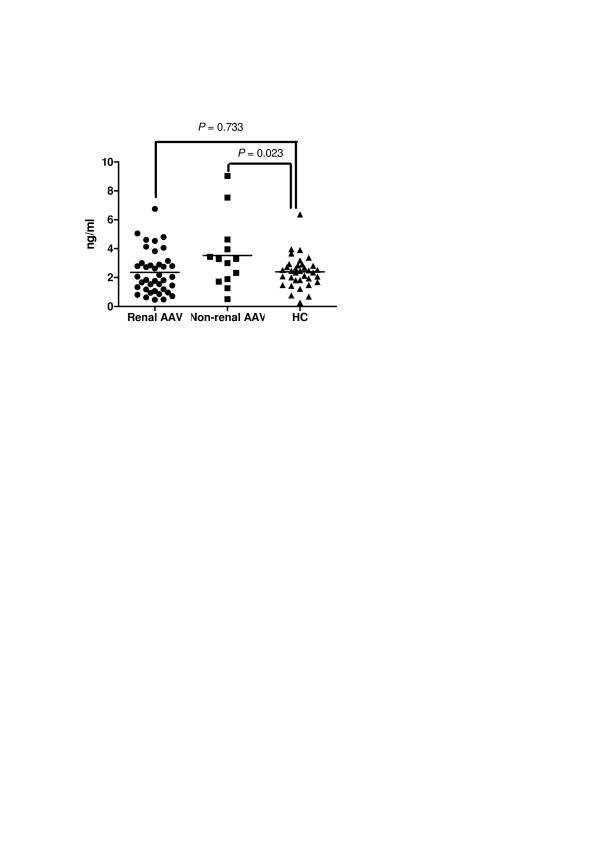
**Baseline HMGB1 levels in AAV patients with renal involvement, without renal involvement and controls**. High-mobility group box 1 (HMGB1) levels at presentation in antineutrophil cytoplasmic antibodies-associated vasculitis (AAV) patients with renal involvement and without renal involvement and in healthy controls (HC) (2.35 ± 1.48 ng/ml vs. 3.52 ± 2.41 ng/ml vs. 2.39 ± 1.09 ng/ml; *P *= 0.046). Using Dunnett's *post-hoc *test, nonrenal AAV patients had higher HMGB1 levels when compared with HC (*P *= 0.023) whereas no difference was found between patients with renal involvement and HC (*P *= 0.733).

Patients without renal involvement (*n *= 13) had increased levels of HMGB1 compared with HC (*P *= 0.023). In contrast, HMBG1 levels in patients with renal involvement (*n *= 39) were no different from those in HC (*P *= 0.733) (Figure 2). No significant difference in mean HMGB1 levels was found between patients with renal involvement who presented granulomatous manifestations in comparison with those without associated granulomatous manifestations (2.34 ± 1.53 ng/ml (*n *= 22) vs. 2.37 ± 1.46 ng/ml (*n *= 17); *P *= 0.826). A negative correlation was observed between serum HMGB1 levels and 24-hour proteinuria (ρ = -0.361, *P *= 0.028) whereas no correlation was found between serum HMGB1 levels and creatinine clearance in AAV patients with renal involvement (*r *= 0.330; *P *= 0.144).

### Longitudinal analysis of HMGB1 levels and relapses

**Figure 3:**
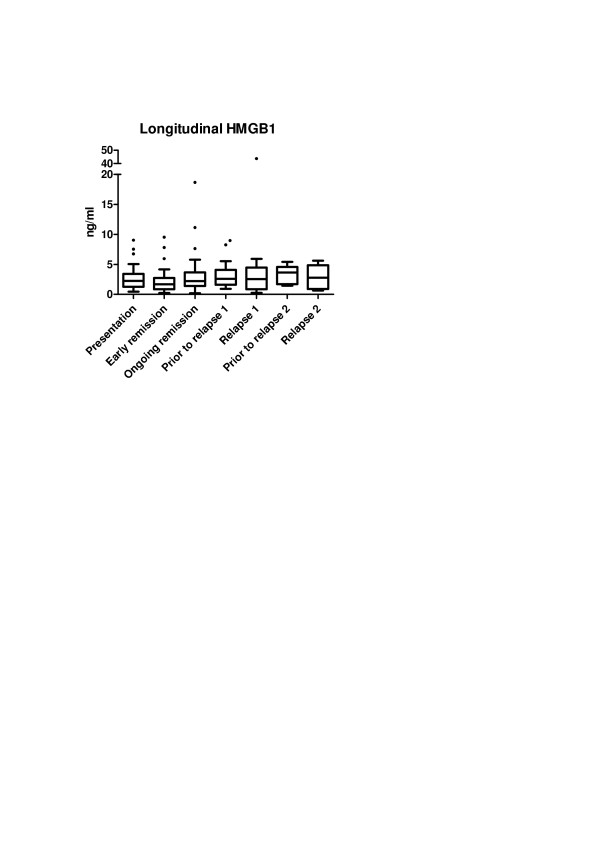
**Longitudinal fluctuation of HMGB1 levels in different phases of the disease in AAV patients**. High-mobility group box 1 (HMGB1) levels decrease significantly from presentation to when remission was induced (*P *= 0.006) but returned to levels similar to baseline at ongoing remission. No significant changes in HMGB1 levels were observed prior to or during disease relapses in antineutrophil cytoplasmic antibodies-associated vasculitis (AAV) patients. Box and whisker plots indicate the 25 to 75% range (boxes), the 5 to 95% range (error bars), and the median value (horizontal lines); dots represent outliers.

**Figure 4:**
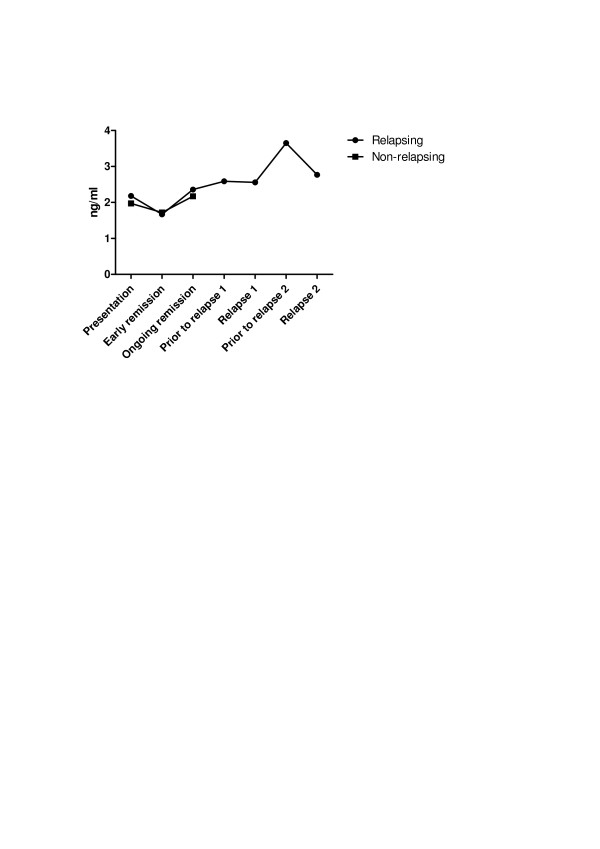
**Comparison of HMGB1 levels during follow-up in relapsing and nonrelapsing AAV patients**. Using a generalized estimating equation model, no significant differences were found between relapsing (*n *= 17) and nonrelapsing (*n *= 32) antineutrophil cytoplasmic antibodies-associated vasculitis (AAV) patients regarding longitudinal levels of high-mobility group box 1 (HMGB1) (*P *= 0.350).

To verify whether HMGB1 levels follow disease activity in AAV, HMGB1 levels were measured at presentation and during the remission period. A significant decrease in median HMGB1 levels (2.35 ng/ml (1.48 to 3.15) vs. 1.69 ng/ml (0.88 to 2.73); *P *= 0.006) was observed at the moment remission had been induced in comparison with baseline but then levels increased significantly again during ongoing remission (1.69 ng/ml (0.88 to 2.73) vs. 2.21 ng/ml (1.42 to 3.68); *P *= 0.004) (Figure 3 and Table 2). During follow-up, at least one disease relapse was observed in 17 AAV patients (34.7%), of whom six patients suffered from two relapses. GPA was the most frequent AAV subset among relapsing patients (82%). No significant differences regarding HMGB1 levels were observed between relapsing and nonrelapsing AAV patients during follow-up (relapsing: 2.18 ng/ml (1.49 to 3.15), 1.67 ng/ml (0.90 to 3.29) and 2.36 (1.33 to 2.75) vs. nonrelapsing: 1.97 ng/ml (1.10 to 3.72), 1.72 ng/ml (0.84 to 2.46) and 2.17 (1.44 to 3.97) at presentation and at remission 3 and 11 months after presentation, respectively; *P *= 0.350) (Figure 4).

**Table 2:** Longitudinal analysis of HMGB1 levels in patients with antineutrophil cytoplasmic antibodies-associated vasculitis

Variable	Presentation	Early remission^a^	Ongoing remission	Prior to relapse 1	Relapse 1	Prior to relapse 2	Relapse 2
HMGB1 (ng/ml)	2.35 (1.48 to 3.15)	1.69* (0.88 to 2.73)	2.21 (1.42 to 3.68)	2.59 (1.61 to 4.09)	2.56 (0.87 to 4.48)	3.65 (1.71 to 4.56)	2.77 (0.91 to 4.86)
Follow-up period (months)	0	3.0^b ^(3.0 to 6.0)	11.0^b ^(6.0 to 12.0)	2.0^c ^(2.0 to 3.0)	20.0^b ^(13.0 to 41.5)	2.0^c ^(1.0 to 2.0)	44.0^b ^(35.2 to 68.5)
BVAS	15.0 (12.0 to 23.5)	0	0	0	12.0 (5.5 to 13.5)	0	11.0 (5.7 to 15.7)
Number of patients	52	49	49	15	17	5	6

Data presented as median (interquartile range). BVAS, Birmingham Vasculitis Activity Score; HMGB1, high-mobility group box 1. ^a^Early remission indicates when remission was achieved following induction treatment. ^b^Months after presentation. ^c^Months prior to the relapse. *Significant *P *value for fluctuation in HMGB1 levels between presentation and early remission.

Aiming to evaluate whether an increase in HMGB1 levels during the remission period could indicate an increased risk of relapse, AAV patients were also evaluated prior to a relapse. However, no significant increase in serum HMGB1 levels could be observed prior to a relapse in comparison with HMGB1 levels during the remission period. Moreover, serum HMGB1 levels prior to a relapse were similar in comparison with HMGB1 levels during the relapses (Figure 3 and Table 2). Serum HMGB1 levels at different time points were not associated with an increased risk of relapse of AAV while fluctuations of serum HMGB1 levels between remission and prior to the first relapse were not associated with an increased risk for relapses (Table 3).

**Table 3:** HMGB1 levels as a biomarker of relapse risk in antineutrophil cytoplasmic antibodies-associated vasculitis patients

HMGB1 levels	Hazard rate	95% confidence interval	*P *value
At baseline	0.812	0.529 to 1.248	0.327
At early remission^a^	0.963	0.641 to 1.446	0.854
At ongoing remission	1.344	0.746 to 2.420	0.325
Prior to relapse 1	0.967	0.776 to 1.205	0.762
Delta between early remission and prior to relapse 1	0.959	0.751 to 1.226	0.739
Delta between ongoing remission and prior to relapse 1	0.919	0.728 to 1.161	0.478

Cox hazard proportional model to evaluate the role of HMGB1 levels as a biomarker of relapse risk in antineutrophil cytoplasmic antibodies-associated vasculitis patients. HMGB1, high-mobility group box 1. ^a^Early remission indicates when remission was achieved.

### Antibodies against HMGB1

Median OD values for anti-HMGB1 antibodies were similar between AAV patients with active disease and HC (0.175 (0.110 to 0.293) vs. 0.254 (0.177 to 0.297); *P *= 0.151). Anti-HMGB1 antibodies were positive in only three out of 24 patients (12.5%) with active AAV, in a median titer (OD: 0.465; range: 0.442 to 0.556) lower than in the two SLE patients included as positive controls (0.592 and 0.659, respectively). Albeit not significant, the AAV patients positive for anti-HMGB1 antibodies had a higher BVAS in comparison with those without anti-HMGB1 antibodies (26.0 (21.5 to 30.5) vs. 12.0 (6.0 to 19.0); *P *= 0.060).

## Discussion

In this study, we evaluated serum HMGB1 levels as a biomarker of disease activity in AAV and investigated anti-HMGB1 antibodies in AAV patients with active disease. We observed that even though a significant correlation was found between HMGB1 and CRP levels at presentation, only AAV patients without active renal disease had significantly higher serum HMGB1 levels than HC. Serum HMGB1 levels decreased significantly in a median of 3 months after presentation but then returned to levels similar to those found at baseline and no significant fluctuation was seen over time in AAV patients, not prior to or during disease relapses. Only a minority of AAV patients with active disease develop anti-HMGB1 antibodies.

Anti-HMGB1 antibodies have been described in sera of patients with septic shock, polymyositis, dermatomyositis and in active SLE [13,20]. In critically ill patients with septic shock, anti-HMGB1 antibodies were associated with a better prognosis and increased survival [21]. In patients with SLE, anti-HMGB1 antibodies were positively correlated with disease activity and anti-double-stranded DNA titers and were negatively correlated with serum complement levels [13,20]. In this study, only a small minority of AAV patients were positive for anti-HMGB1 antibodies. Moreover, no significant association between anti-HMGB1 antibodies and BVAS score could be observed. A previous study also failed to demonstrate anti-HMGB1 antibodies in 22 patients with AAV and active renal involvement [22].

The technique used to measure serum HMGB1 levels is a relevant issue because serum HMGB1 levels are usually five times higher when a western blot technique is used in comparison with ELISA, although both assays correlate well [23]. The binding of HMGB1 to different serum/plasma molecules, especially IgG_1_, interferes with its detection by ELISA systems, which is considered the main reason for this discrepancy [24]. Moreover, the depletion of IgG from the sera of SLE patients lowered serum HMGB1 levels detected by ELISA, indicating that this interference might in part be due to anti-HMGB1 antibodies in SLE [13]. We therefore firstly tested anti-HMGB1 antibodies in AAV patients before choosing the technique to measure serum HMGB1. Since only a few AAV patients presented anti-HMGB1 antibodies, the ELISA technique was used to measure serum HMGB1 in the present study. Nevertheless, current ELISA methods seem to have limitations in assessing HMGB1 levels as a surrogate marker of active disease in different scenarios due to potential interference in its detection by serum factors, including anti-HMGB1 antibodies. Perhaps only free HMGB1 could be detected by current ELISA systems instead of total HMGB1 [24,25]. Furthermore, it is now known that functionality of HMGB1 is affected by the redox state of its three cysteine residues (C23, C45 and C106) and future methods to detect HMGB1 should take this functional nuance into account. The all-thiol form of HMGB1 has only chemotactic activity while disulfide-bonded HMGB1 (between C23 and C45) induces cytokine release through binding of TLR-4. No cytokine-stimulating or chemotactic activity is found in the fully oxidized HMGB1 [26,27].

We observed that AAV patients with renal involvement presented similar HMGB1 levels in comparison with HC while AAV patients without active nephritis had significantly higher HMGB1 levels than HC. We therefore speculate that the reason for finding serum HMGB1 levels in AAV patients at presentation similar to HC, in contrast to other studies evaluating HMGB1 in AAV [15,17], could be the high number of patients with active renal involvement evaluated in the present study (75.0%). Although Bruchfeld and colleagues have previously described higher serum HMGB1 levels in AAV patients with biopsy-proven active nephritis in comparison with patients without active renal inflammation, no systematic comparison was made with AAV patients presenting active disease in other organs or systems [16]. In line with our results, Henes and colleagues described lower serum HMGB1 levels in GPA patients with predominantly vasculitic manifestations when compared with those with predominantly granulomatous manifestations. Active nephritis was the most frequent feature observed among GPA patients with vasculitic manifestations. Granulomatous inflammation may have contributed to higher HMGB1 levels in GPA patients with granulomatous manifestations [17]. In our study, patients with GPA and localized disease presented higher levels of HMGB1 at baseline than patients with generalized disease. Also, AAV patients with pulmonary nodules and/or lung infiltrates had higher levels of HMGB1 than those with alveolar hemorrhage. Differences, however, were not significant in both situations. Nonetheless, the presence of granulomatous manifestations in AAV patients did not seem to influence HMGB1 levels in AAV patients with simultaneously active nephritis, indicating that HMGB1 levels were mostly influenced by renal involvement. The increased expression of HMGB1 in renal tissue [16] and the necrotizing nature of glomerulonephritis in AAV indicate that both active release by activated cells and passive release by dying necrotic cells could be the source of extracellular HMGB1 in renal involvement of AAV besides systemic inflammation. However, whether leakage of HMGB1 into urine due to renal damage contributes to lower serum HMGB1 levels in parallel with increased urinary levels of HMGB1 in active glomerulonephritis in AAV is still unknown.

Serum HMGB1 levels have been correlated with disease activity in AAV in cross-sectional studies. Nevertheless, longitudinal evaluation of serum HMGB1 levels demonstrated only a significant decrease in HMGB1 levels approximately 3 months after presentation. Thereafter, with ongoing remission, serum HMGB1 returned to levels similar to those found at presentation. Serum HMGB1 levels prior to relapses and during relapses were somewhat higher than baseline levels but this difference was not significant. Achievement of disease remission in response to sustained immunosuppressive therapy may be the reason for lower HMGB1 levels within 3 months after disease presentation. Although approximately one-half of AAV patients at presentation were already under immunosuppressive therapy for a median 3 weeks and no difference in serum HMGB1 levels could be found between patients with and without therapy, both treated patients and untreated patients had a similar median BVAS at baseline evaluation. After achieving remission most AAV patients were still on oral prednisolone and cyclophosphamide, whereas during ongoing remission immunosuppressive therapy was tapered and cyclophosphamide was changed to azathioprine. Prior to relapses in the majority of AAV patients, immunosuppressive therapy was withdrawn (data not shown). This reduction in immunosuppressive therapy may therefore account for the significant increase in serum HMGB1 levels from early remission to ongoing remission.

In this study, no difference regarding serum HMGB1 levels could be found during follow-up between relapsing and nonrelapsing AAV patients and fluctuations of serum HMGB1 levels during the remission period or prior to a relapse were not associated with an increased risk of relapses in AAV. Hence, fluctuations of serum HMGB1 levels cannot be used as a surrogate marker for disease activity in AAV.

## Conclusions

Nonrenal AAV is associated with higher serum HMGB1 levels at presentation and serum HMGB1 levels decrease significantly within 3 months after presentation, possibly due to immunosuppressive treatment, but during ongoing remission HMGB1 levels return to levels similar to those observed at presentation. A slight nonsignificant increase in HMGB1 levels is observed prior to and during relapses in comparison with baseline levels. No association between fluctuations of serum HMGB1 levels and risk of relapse could be found. Circulating HMGB1 measured by ELISA therefore does not seem to be a useful biomarker of disease activity in AAV. Patients with AAV did not develop significant anti-HMGB1 antibodies during active disease.

## Abbreviations

AAV: antineutrophil cytoplasmic antibodies-associated vasculitis; ANCA: antineutrophil cytoplasmic antibodies; BVAS: Birmingham Vasculitis Activity Score; CRP: C-reactive protein; ELISA: enzyme-linked immunosorbent assay; GPA: granulomatosis with polyangiitis; HC: healthy controls; HMGB1: high-mobility group box 1; MPA: microscopic polyangiitis; OD: optical density; PR3: proteinase 3; RLV: renal limited vasculitis; SLE: systemic lupus erythematosus; TLR: toll-like receptor.

## Competing interests

The authors declare that they have no competing interests.

## Authors' contributions

AWSdS contributed to the study design, collected data from patients' medical records, performed laboratory tests, conducted the statistical analysis and drafted the manuscript. JW contributed to the study design, performed laboratory tests and revised the manuscript. JB contributed to the study design, performed laboratory tests, helped the interpretation of results and revised the manuscript. PCL contributed to the study design, helped the interpretation of data and revised the manuscript. CAS participated in the acquisition of data, interpreted results and revised the manuscript. MB conceived the study, contributed to the study design, interpretation of data and revised the manuscript. CGMK conceived the study, contributed to the study design and interpretation of data and revised the manuscript.
